# Deep forecasting of translational impact in medical research

**DOI:** 10.1016/j.patter.2022.100483

**Published:** 2022-04-08

**Authors:** Amy P.K. Nelson, Robert J. Gray, James K. Ruffle, Henry C. Watkins, Daniel Herron, Nick Sorros, Danil Mikhailov, M. Jorge Cardoso, Sebastien Ourselin, Nick McNally, Bryan Williams, Geraint E. Rees, Parashkev Nachev

**Affiliations:** 1 High Dimensional Neurology Group, UCL Queen Square Institute of Neurology, University College London, Russell Square House, Bloomsbury, London WC1B 5EH, UK; 2 Research & Development, NIHR University College London Hospitals Biomedical Research Centre, London WC1E 6BT, UK; 3 Wellcome Data Labs, Wellcome Trust, London NW1 2BE, UK; 4 School of Biomedical Engineering & Imaging Sciences, King’s College London, London WC2R 2LS, UK; 5 UCL Institute of Cardiovascular Sciences, University College London, London WC1E 6BT, UK; 6 Faculty of Life Sciences, University College London, Gower Street, London WC1E 6BT, UK

**Keywords:** deep learning, representation learning, natural language processing, research impact, translational research

## Abstract

The value of biomedical research—a $1.7 trillion annual investment—is ultimately determined by its downstream, real-world impact, whose predictability from simple citation metrics remains unquantified. Here we sought to determine the comparative predictability of future real-world translation—as indexed by inclusion in patents, guidelines, or policy documents—from complex models of title/abstract-level content versus citations and metadata alone. We quantify predictive performance out of sample, ahead of time, across major domains, using the entire corpus of biomedical research captured by Microsoft Academic Graph from 1990–2019, encompassing 43.3 million papers. We show that citations are only moderately predictive of translational impact. In contrast, high-dimensional models of titles, abstracts, and metadata exhibit high fidelity (area under the receiver operating curve [AUROC] > 0.9), generalize across time and domain, and transfer to recognizing papers of Nobel laureates. We argue that content-based impact models are superior to conventional, citation-based measures and sustain a stronger evidence-based claim to the objective measurement of translational potential.

## Highlights

• Deep learning models of biomedical paper content can accurately predict translation
• Deep content models substantially outperform traditional citation metrics
• Models trained on patent inclusion transfer to predicting Nobel Prize-preceding papers
• Science policy is potentially better informed by deep content models than by citations

## The bigger picture

The relationship of scientific activity to real-world impact is hard to describe and even harder to quantify. Analyzing 43.3 million biomedical papers from 1990–2019, we show that deep learning models of publication, title, and abstract content can predict inclusion of a scientific paper in a patent, guideline, or policy document. We show that the best of these models, incorporating the richest information, substantially outperforms traditional metrics of paper success—citations per year—and transfers to the task of predicting Nobel Prize-preceding papers. If judgments of the translational potential of science are to be based on objective metrics, then complex models of paper content should be preferred over citations. Our approach is naturally extensible to richer scientific content and diverse measures of impact. Its wider application could maximize the real-world benefits of scientific activity in the biomedical realm and beyond.

## Teaser

Analyzing 43.3 million biomedical papers from 1990–2019, we show that deep learning models of publication title and abstract content can predict inclusion in a patent, guideline, or policy document with far greater fidelity than citation metrics alone. If judgments of the translational potential of science are to be based on objective metrics, then complex models of paper content should be preferred over citations.

## Introduction

Scientometrics has existed for only a small fraction of the history of science itself, sparked by the logical empiricists of the Vienna Circle in their philosophical quest to construct a unified language of science.^1^ Developed into the familiar, citation-centered form through arduous manual extraction in the mid-20th century,^2^^,^^3^ its indicators have proliferated in the Internet age. They now dominate the research landscape, routinely informing major funding decisions and academic staff recruitment worldwide.^4^, ^5^, ^6^, ^7^, ^8^

The importance of the original goal has become magnified over time: to measure scientific progress regardless of funding or ideology, uncolored by the reputations of individuals or institutions. But the fundamental focus of its current solution—the volume and density of discussion in print—is detached from the ultimate, real-world objective and subject to familiar distortions, such as the popularity of papers notable only for being spectacularly wrong.^9^, ^10^, ^11^

These concerns are amplified in medical science, whose primary focus is not merely knowledge but impact on patient health: necessarily a consequence rather than a constitutive characteristic of research activity, neither easily benchmarked nor directly optimized. And there is no doubt that optimization is needed; over the past 60 years, the number of new drug approvals per unit R&D spend has consistently halved every 9 years, whereas published medical research has doubled with the same periodicity,^12^ and only 0.004% of basic research findings ultimately lead to clinically useful treatments.^13^ The critical pre-requisite for all research—funding—shows substantial randomness in its distribution,^14^ enough for at least one major healthcare funder to award grants by lottery.^15^ Any decision function based on random chance, or a process demonstrably not much better than random chance, leaves room for improvement, particularly when commanding approximately $1.7 trillion global annual investment across the United States, Japan, South Korea, and the European Union.^16^

Is this state of affairs partially caused by over-reliance on misleading scientometrics, have we simply not found the right metrics yet, or is the relation between scientific activity and consequent impact opaque to objective analysis? To address these crucial questions, we need a fully inclusive survey of published medical research that relates its characteristics to an independently measured translational outcome as close to real-world impact as can be quantified. This relationship must be explored with models of sufficient expressivity to detect complex relations between many candidate predictive factors beyond paper-to-paper citations. The extant literature is largely limited to modeling keywords or simple representations of semantic content,^17^, ^18^, ^19^, ^20^, ^21^ within specific subdomains, or comparatively restricted bibliographic databases,^22^, ^23^, ^24^, ^25^, ^26^ and without exploration of the impact of data dimensionality and model flexibility.

Here we provide the first comprehensive, field-wide analysis of translational impact measured by its most widely accepted proximal indices—patents, guidelines, or policies—based on 29 years of data from the medical field encompassing 43.3 million published papers. We quantify the ability to predict inclusion in future patents, guidelines, or policies from conventional age-normalized citation counts and compare this with the predictive fidelity of deep learning models incorporating more complex features extracted from metadata, titles, and abstracts. We evaluate the performance of the best model across time and thematic domain and in transfer to the task of recognizing papers of Nobel laureates. We derive succinct, surveyable representations of paper title and abstract content with deep autoencoding of transformer-based text embeddings and of publication metadata with stochastic block models. The breadth and depth of analysis allow us to draw strong conclusions about the comparative fidelity of conventional bibliographic and novel semantic predictors of translational impact, with substantial implications for research policy.

## Results

### Citations

**Figure 1:**
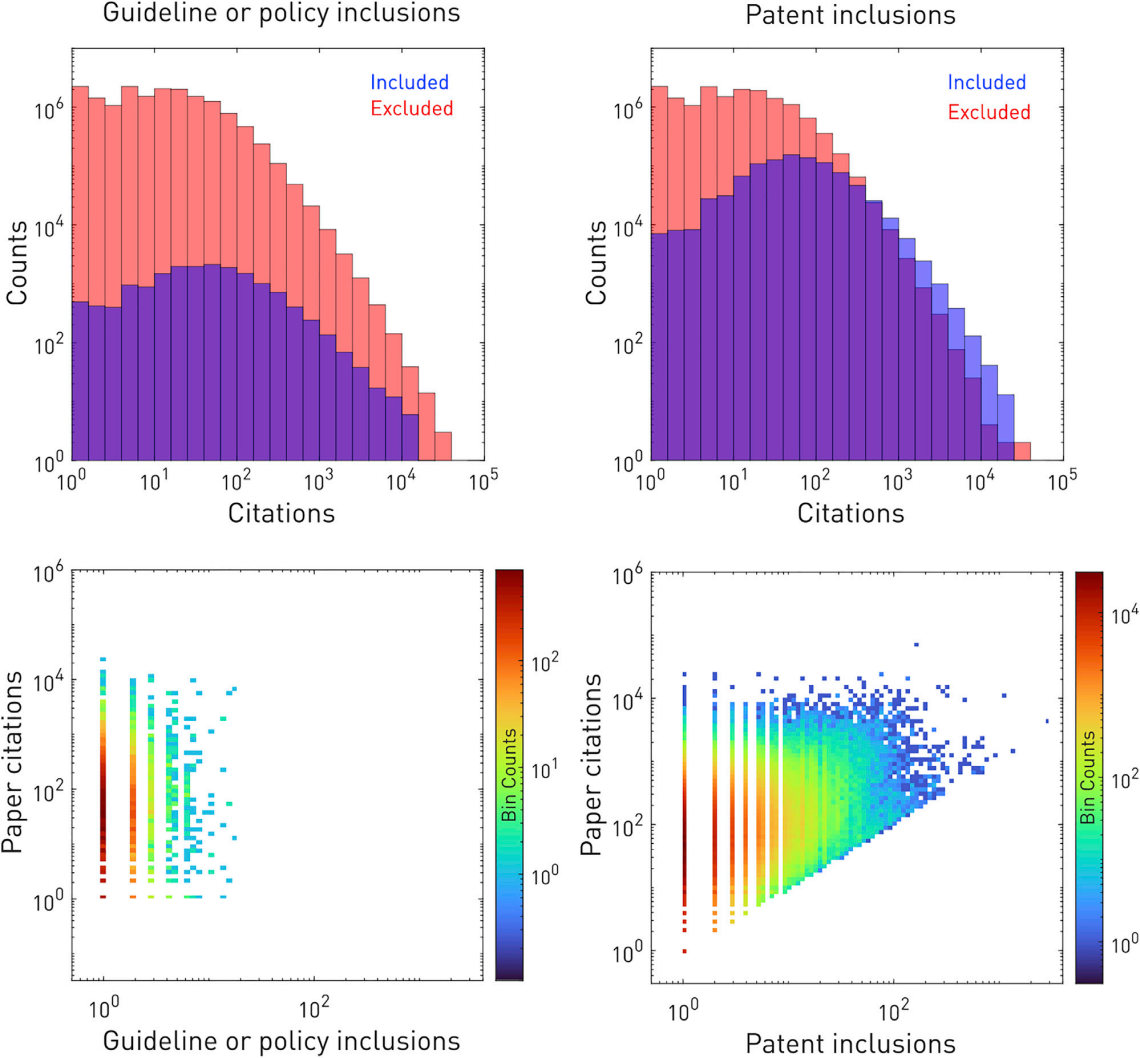
Relationship between paper citations and translational document inclusions Shown are citation histograms of papers included (red) or not included (blue), plotted with semi-transparency on the same log axes, in guideline or policy documents (top left) or patents (top right). The area of overlap is shown in purple, and the contrasting papers are all other identically filtered biomedical papers with at least one citation.. The relationship between citation and inclusion counts for included papers is shown in binned scatterplots for guideline or policy inclusions (bottom left) and patent inclusions (bottom right), also plotted on log axes.

Over the period from January 1990 to March 2019, only 17.1 million of the 43.3 million published papers categorized as medical by Microsoft Academic Graph were cited at least once. Of these, 964,403 were included in a patent and 16,752 in a guideline or a policy document. Included papers were more frequently cited, but the numbers of citations and inclusions were weakly correlated (Pearson’s r = 0.094 for guidelines or policies, r = 0.248 for patents; Figure 1). The mean time delay from paper publication to first patent inclusion was 4.73 years (SD 4.54; Figure S1).

### Predictive performance

A series of models was developed to investigate the relative contribution of three data modalities—annual paper citations, metadata only, and the combination of metadata and abstract/title embeddings—in predicting two translational outcomes: a paper’s inclusion in a patent or policy/guideline reference list. Attempting to predict inclusion in a guideline or policy document from the traditional measure of impact—annual paper citations—yielded a mean cross-validated area under the receiver operating curve (AUROC) of 0.766 with univariable logistic regression (Citations-LogisticRegression) and 0.767 with an optimized univariable multilayer perceptron (MLP) model (Citations-MLP).

**Figure 2:**
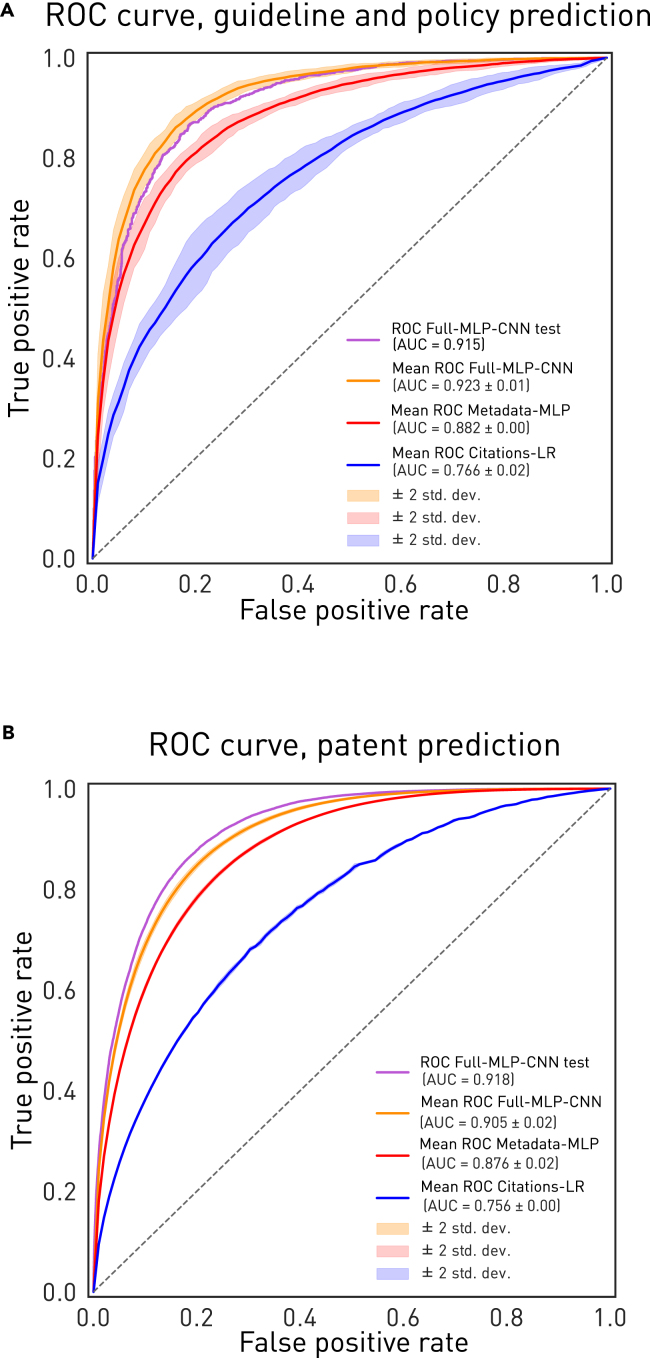
Model predictive performance (A and B) Shown are cross-validated receiver operating curves (ROCs) for the Full-NLP-CNN model trained on metadata and title and abstract embeddings (orange for validation, purple for held-out test) and the Metadata-MLP model (red) and the Citation-LogisticRegression model trained on citation count per year (blue) for guideline or policy inclusions (A) and patent inclusions (B). The confidence intervals are ±2 SD on cross-validation.

In contrast, a high-dimensional model trained on metadata and title and abstract embeddings, based on a hybrid MLP and convolutional neural network (CNN), Full-MLP-CNN, achieved an AUROC of 0.915 and average precision (AP) of 0.919 on unseen test data (Figure 2A). The MLP trained on only metadata, without title or abstract embeddings (Metadata-MLP), achieved a lower mean cross-validated AUROC of 0.882, significantly so, as judged by cross-validation confidence intervals.

For the task of predicting patent inclusions, annual paper citations yielded a mean cross-validated AUROC of 0.756 with univariable logistic regression (Citations-LogisticRegression) and optimized univariable MLP (Citations-MLP).

A high-dimensional model trained on metadata and title and abstract embeddings (Full-MLP-CNN) achieved a much higher AUROC of 0.918 and AP of 0.859 on unseen test data (Figure 2B). The MLP trained only on metadata, without title or abstract embeddings (Metadata-MLP), achieved a lower mean cross-validated AUROC of 0.876.

Across both tasks, a high-dimensional neural network model trained on metadata and content embeddings substantially outperforms more commonly used citation based metrics when predicting future translational impact.

### Performance over time and across research domain

**Figure 3:**
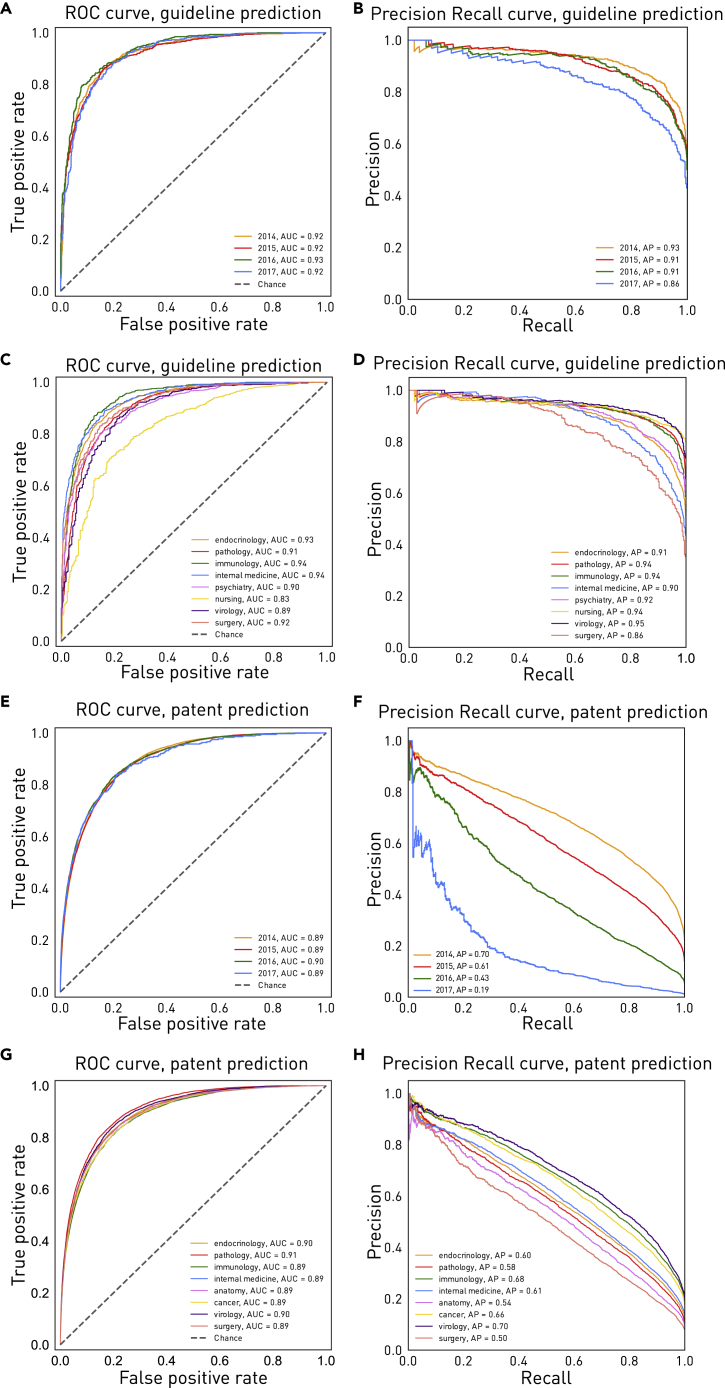
Predictive performance in future years and most common fields (A–H) ROC curves (A and C) and precision-recall curves (B and D) for the Full-MLP-CNN model trained on data from 1990–2013 and tested on papers published in the subsequent 4 years, plotted by year, for guideline or policy inclusions and patent inclusions, respectively. Also shown are ROC curves (E and G), and precision-recall curves (F and H) for the Full-MLP-CNN model trained on data from 1990–2013 and tested on data from 2014–2019, plotted by each of the top 8 most common fields, for guideline or policy inclusions and patent inclusions, respectively.

To test the generalizability of the models, we must examine sustained performance over time and across domains. For guideline or policy documents, the high-dimensional Full-MLP-CNN model trained only on data from 1990–2013 and tested on out-of-sample papers published over all succeeding 4 years achieved an AUROC of 0.920 and an AP of 0.911 (non-averaged). Crucially, there was no appreciable diminution in fidelity over time for individual years (Figures 3A, 3B, and S2A). Performance was consistently good to excellent within each of the top 8 most common domains of medicine (Figures 3C and 3D).

An identical analysis of patent inclusions produced a similar picture, yielding an AUROC of 0.902 and an AP of 0.606 for out-of-sample papers published over all succeeding 4 years (non-averaged), with no diminution over time for individual years on AUROC but some diminution on AP (Figures 3E and 3F), likely reflecting the correspondingly shorter time frames for realization of any patent inclusion; papers in later years would have to be included within fewer years, leading to an artificially deflated proportion of included papers and penalized specificity. Indeed, the AUROC improved with time (Figure S2B), likely reflecting geometric growth in publication numbers (doubling every 9 years), changes in publication and citation patterns, and an increase over time of patents in which papers may be included. Future performance was consistently good to excellent within each of the top 8 most common domains (Figures 3G and 3H).

### Data ablation studies

Rebuilding the Full-MLP-CNN models without paper-level metrics—paper citations, paper rank, and paper mentions—and separately, also without attributes influenced by factors of merit extrinsic to the paper itself—affiliation, authors, journal, and field—yielded slightly diminished fidelity. For guideline and policy inclusions, the paper-level metrics-ablated model achieved an AUROC of 0.905, and the model ablated of paper-level metrics and extrinsic factors achieved an AUROC of 0.896. The corresponding values for models based on metadata only were 0.832 and 0.816. A model trained only on title-abstract embeddings, without any metadata at all, achieved an AUROC of 0.892 (Figure S3A). For patent inclusions, identically constrained models yielded AUCROCs of 0.881, 0.866, 0.847, 0.813, and 0.822, respectively (Figure S3B).

### Transfer to predicting papers preceding a Nobel Prize

If the high-dimensional models are capable of capturing fundamental features of translational impact, they may identify papers whose impact is judged by other criteria. To test for such transfer learning, we applied our best patent model (Full-MLP-CNN)—trained on data with Nobel Prize-preceding papers removed and without retraining on new targets—to the task of identifying the papers, published before the prize was awarded, of Nobel laureates in physiology or medicine from 1990–2019.

We identified 166 papers, 60 of which were included in patents. Strikingly, the Full-MLP-CNN model retrieved a substantially higher proportion of Nobel laureate papers (103 of 166) than Metadata-MLP (86 of 166) or Citations-LogisticRegression (23 of 166) while retaining superior fidelity for detecting patent inclusions (AUROC 0.79 versus 0.73 and 0.73, respectively).

### Predictors of inclusion

A complex, high-dimensional model cannot easily yield intelligible weightings of predictive importance because its decision is a highly non-linear function of a large set of input features. A coarse indication of relative feature importance can nonetheless be derived from alternative architectures of lesser flexibility. Here a boosted trees model (Metadata-AdaBoost) was used, trained on the metadata and optimized by grid search to similar performance as the MLP (AUROC 0.878, guideline or policy inclusions; AUROC 0.877, patent inclusions) (Tables S1A and S1B).

For guideline or policy inclusions, the rank of the paper, a metric provided by Microsoft Academic Graph (MAG),^27^ reflecting the eigencentrality-based “influence” of a paper, had the highest feature importance, followed by the paper count, citation count, and rank of the journal in which the paper was published. For patent inclusions, the top three features were related to journal productivity-related metrics. The top 10 feature importances of models restricted to data before 2014 were very similar to those trained on the full time period, although the ordering was different in the patent model, with greater weight on citations, year, and field productivity (Table S1B).

### Deep semantic structure of titles and abstracts

**Figure 4:**
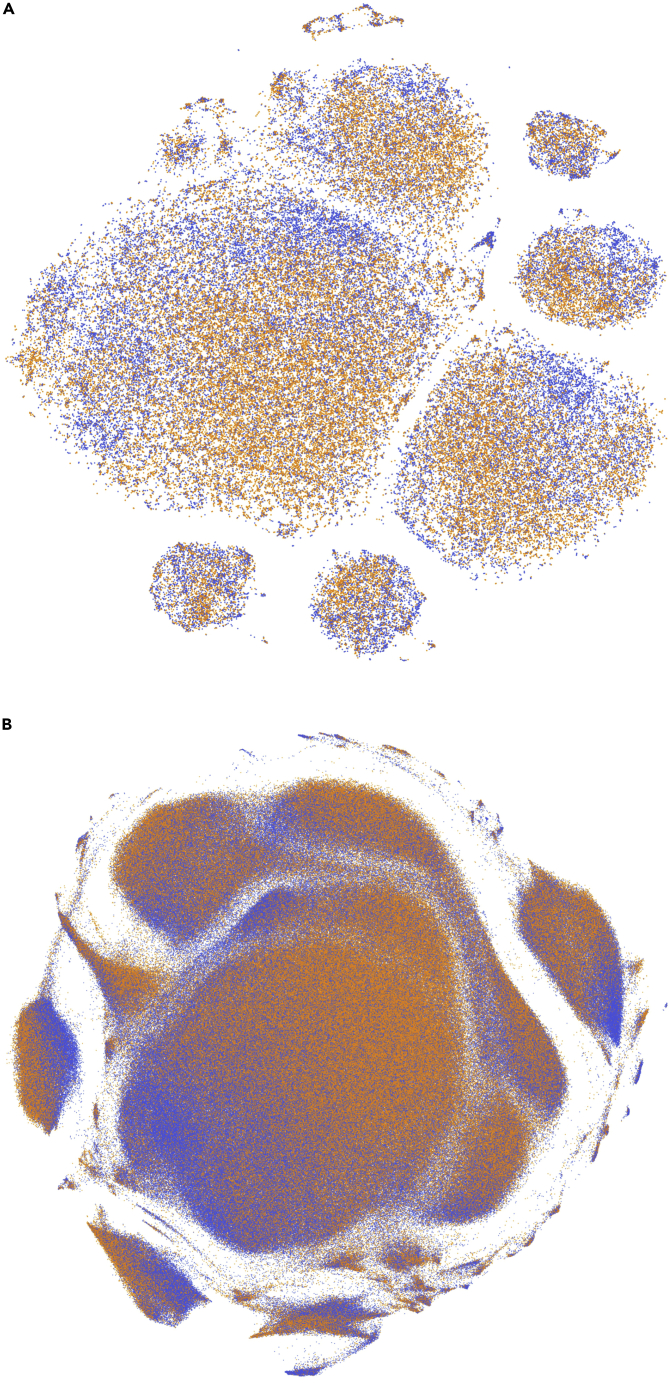
t-distributed stochastic neighbor embedding (t-SNE) projection in 2 dimensions of title and abstract BioBERT autoencoder embeddings (A and B) Labeled by the presence (orange) or absence (blue) of a guideline or policy (A) or patent (B) inclusion. Note the discernible data structure that enables accurate prediction of inclusion but is too complex to be reduced to any small set of characteristic features.

Textual analysis of title or abstract content cannot easily yield an intelligible set of predictive features as in the foregoing models. But we can visualize the sentence-level embeddings of the title and abstract encoded by BioBERT,^28^ a rich, context-aware representation of natural language concepts tuned on biological text, through a succinct representation generated by a deep autoencoder. Represented in a two-dimensional space through non-linear dimensionality reduction, the embeddings showed a degree of disentanglement of clusters rich in guideline or policy inclusions versus none (Figure 4A). This reveals intrinsic structure in the data exploited by the hybrid model to achieve the high classification performance observed. An identical analysis of the structure of the patent inclusion embeddings revealed a similar intrinsic structure (Figure 4B).

### Graph community structure

The similarity and dissimilarity between papers can be modeled as a graph whose edges index the dependencies between individual features. Hierarchically arranged distinct patterns of similarity, the graph’s community structure, can then be revealed by stochastic block modeling,^29^ here performed separately for guideline or policy-included papers and patent-included papers, each compared against all other papers.

**Figure 5:**
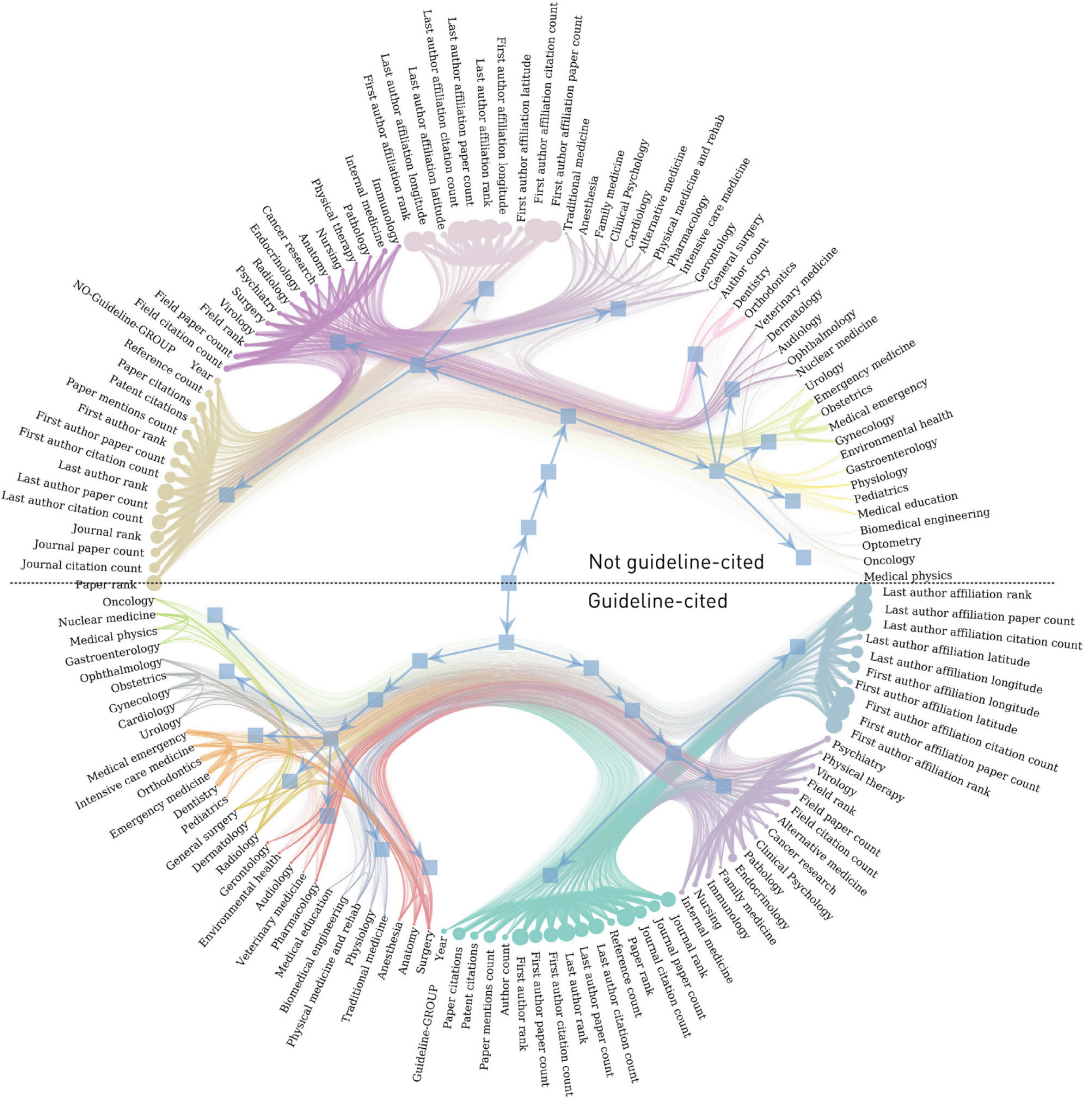
Nested stochastic block models (SBMs) showing the community structure of the metadata of papers included in guidelines or policy versus those not included Node size in these models corresponds to the eigencentrality of each feature, edge weight corresponds to the pairwise absolute value of the correlation coefficient between features, and the colours indicate community membership at the lowest hierarchy. The included class is the bottom hemifield.

**Figure 6:**
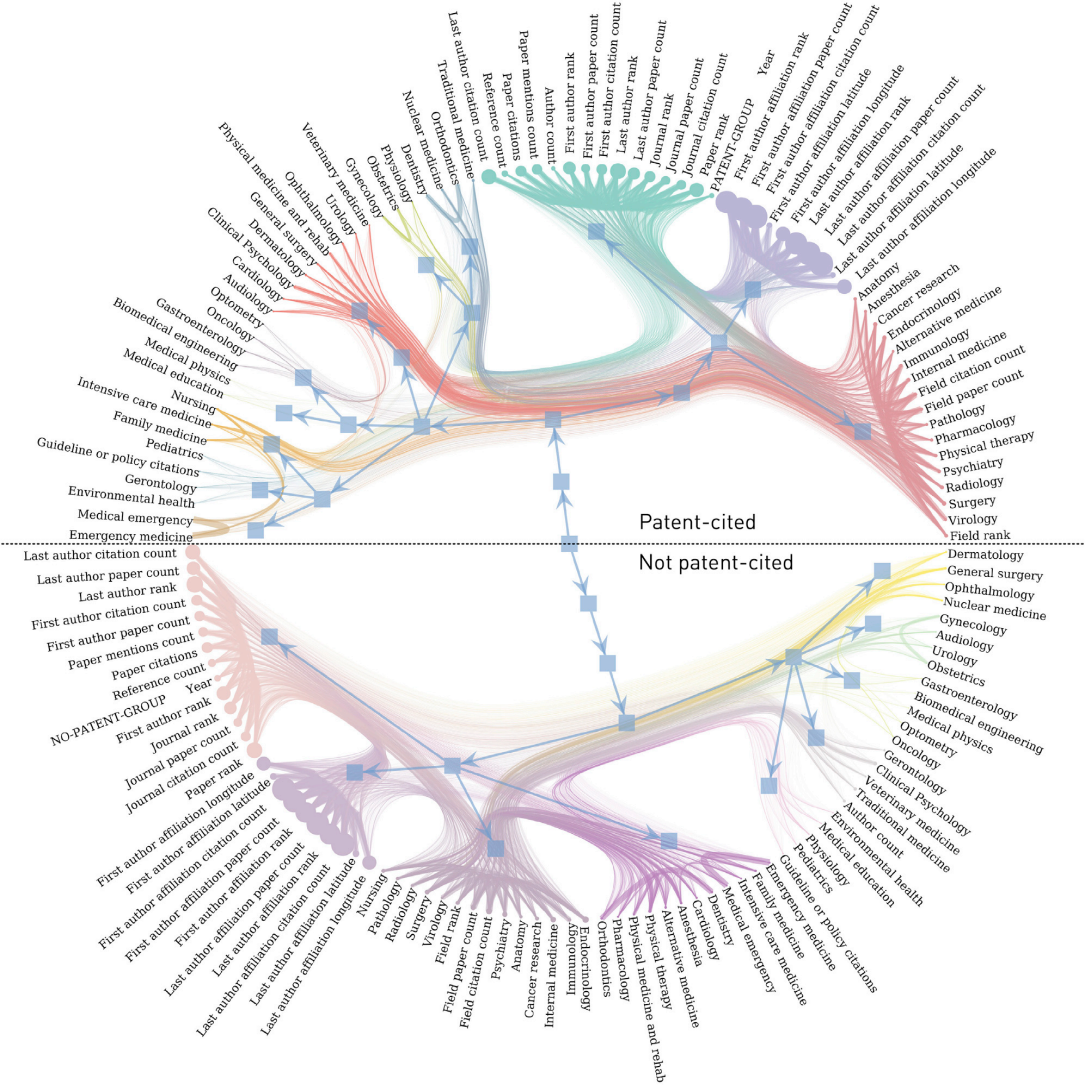
Nested SBMs showing the community structure of the metadata of papers included in patents versus those not included Node size in these models corresponds to the eigencentrality of each feature, edge weight corresponds to the pairwise absolute value of the correlation coefficient between features, and the colours indicate community membership at the lowest hierarchy. The included class is the top hemifield.

Distinct communities of author, institutional, journal, and domain features emerged across both groups (Figures 5 and 6). Overall, the community structures of papers not included in guideline, policy or patent documents were most similar, as indexed by pairwise comparisons of the log-normalized mutual information of the inferred model parameters, and the community structure of guideline- or policy-included papers was most distinctive (Figure S4). This observation cohered with the structure of an undirected features graph, weighted by the absolute correlation coefficient between features, that showed patent inclusions to be more centrally embedded within the wider network of metadata than guideline or policy inclusions (Figure S5).

Contrasting the effect of inclusion in the guideline or policy group, indices related to the first author and journal were more decisive in the included papers, whereas indices related to the institution and journal were more decisive in the others (Figure S6A). The domains of virology, endocrinology, alternative medicine, psychiatry, nursing, and environmental health were also more prominent in the former and surgery, radiology, traditional medicine, and rehabilitation in the latter. The effect of inclusion in the patent group was most strongly manifested in institutional indices for the included group and field indices for the others. The contrast between domains was more striking than in the guideline or policy model, with pharmacology especially dominant in the included group and general medical specialties in the others (Figure S6B).

### The translational impact of journals

Journal impact factors—indices of the annual citation return of an average paper—exclude patent, guideline, or policy inclusions. So ranked, the top 10 journals in the medical domain based on cited papers published between 1990 and 2019 are listed in Table 1. This corresponds to a medical domain “impact factor” over three decades rather than the commonly reported annual. The equivalent ranking for guideline and patent inclusions, identically filtered, are listed in Tables 2 and 3, respectively. In the absence of plausibly objective weighting of policies or guidelines, this metric will be sensitive to the numerosity of distinct policy documents within any given domain, reflecting its political, regulatory, or administrative complexity.

**Table 1:** Top 10 journals by paper citations per paper

Journal	Paper citations/total papers	Paper citations	Total papers
Annual Review of Immunology	480.835	376,494	783
Physiological Reviews	407.457	392,789	964
Annual Review of Neuroscience	383.877	240,691	627
Psychological Bulletin	333.302	409,295	1,228
Pharmacological Reviews	318.500	210,847	662
Cell	295.994	3.229.298	10,910
Annual Review of Psychology	283.465	161,575	570
CA: A Cancer Journal for Clinicians	263.673	240,733	913
Psychological Review	262.322	247,107	942
Clinical Microbiology Reviews	250.334	239,319	956

**Table 2:** Top 10 journals by guideline or policy inclusions per paper

Journal	Guideline or policy inclusions/total papers	Guideline or policy inclusions	Total papers
Tobacco Control	0.094	3,120	3,325
Eastern Mediterranean Health Journal	0.091	282	3,090
Noise & Health	0.085	55	649
Human Resources for Health	0.084	69	824
Health Policy and Planning	0.079	157	1977
Influenza and Other Respiratory Viruses	0.061	63	1,036
Globalization and Health	0.043	29	681
PLOS Medicine	0.040	152	3,809
Bulletin of The World Health Organization	0.039	247	6,278
Trauma, Violence, & Abuse	0.038	22	574

**Table 3:** Top 10 journals by patent inclusions per paper

Journal	Patent inclusions/total papers	Patent inclusions	Total papers
Annual Review of Immunology	5.733	4,489	783
Nature Biotechnology	3.862	17,042	4,413
Protein Engineering	3.153	2,557	811
Pharmacological Reviews	3.045	2,016	662
Trends in Biotechnology	2.990	4,733	1,583
Cell	2.725	29,729	10,910
Journal of Experimental Medicine	2.293	24,105	10,513
Advanced Drug Delivery Reviews	2.167	6,862	3,166
Chemical Reviews	1.925	1,305	678
Transfusion Science	1.845	1,552	841

## Discussion

We provide the first comprehensive framework for forecasting the translation of published medical research in the form of patent, guideline or policy inclusions, reveal the community structure of translational inclusions, and compute the top translationally relevant journals across biomedicine over the past three decades.

### Simple citation versus complex content metrics

We show that standard citation metrics are markedly inferior to those derived from complex models based on more detailed descriptions of published research. If objective metrics are to be used in translational assessment, then the use of conventional metrics is here shown to be insupportable. Our analysis suggests that the problem rests not with citations but with the expressivity of any simple metric of something as constitutionally complex as research translation. It is clear that the translational signal is distributed through the combinatorial fabric of paper citation networks, metadata, and content captured in titles and abstracts. No easily interpretable scalar value could capture it. Conversely, that surprisingly economical information about a paper—its metadata, title, and abstract—can be exploited by the right modeling architecture to yield high predictive fidelity means that no one could argue that no objective alternative is available. Even without full text information, we can confidently identify large swathes of research activity unlikely to inform guidelines or policy or to become the substrate of patents across time and diverse subdomains. The choice now is not between subjective, qualitative assessment and simple quantitative metrics, but includes machine learning models that are no less objective, reproducible, and generalizable for being complex.

We cannot and do not argue that machine learning models remove the need for qualitative assessment, but only that the quantitative metrics in current use could be far better. Content parameterization of entire scientific fields, limited in existing literature to keyword analysis or word-level or simple document-level embeddings,^17^, ^18^, ^19^, ^20^, ^21^ can be usefully extended using deep learning models, such as those applied here, to capture a greater depth of meaning from abstracts. Indeed, the clearly observed relation between model complexity and achieved fidelity suggests that modeling of the body of a paper—currently infeasible for copyright reasons—is likely to yield still higher fidelity. The analysis of metadata may additionally be expanded with inclusion of wider dissemination scores, such as those captured in Altmetric, which has already been examined at single-journal scale,^25^ and more widely across the full Scopus database,^22^ for predicting paper-paper citations. This will inevitably usher an examination of policies on the right trade-off between performance and intelligibility that must be settled politically, not empirically.

### Possibilities and limitations of complex translational forecasting

Our models are of direct, first-order inclusions, indifferent to the upstream published sources a given paper itself cites. They may be more likely to predict the translational potential of a meta-analysis, for example, than that of any of the preceding studies informing it. But the proposed framework can be naturally extended to second- or higher-order inclusions earlier in the citation path, weighting the cascade of information down the full translational pathway in a principled way. For example, the citation nexus has been modeled as a graph,^30^ with publication-based metrics as the predictive target, in evolution of established approaches for predictive modeling of bibliographically defined impact.^31^^,^^32^ The constraint on inclusion depth, among other considerations, prevents naive use of our models to determine the causal sufficiency of translation, but no one would claim that any metric within so complex a system could plausibly index causality on its own. A complex model can also be used to distinguish empirical from meta-analytical papers with potentially greater accuracy than bibliographic “article type” tags, weighting inclusions by their empirical content.

Equally, although unethical biases can corrupt carelessly designed or interpreted complex models, they can also be revealed by them, where the neglected subpopulation is defined by the complex interaction of several variables of ethical concern simple models are too crude to illuminate. Insisting on simple, low-dimensional decision boundaries does not remove bias but merely conceals it from view; complex models, correctly designed and used, are not the problem here but an essential part of the solution. A sharp distinction must be drawn between simplicity and explainability; where a system is inherently, irreducibly complex, a simple metric cannot be explanatory. The unprecedented scale of analyzed data, drawn from the largest open bibliographic repository in the world, limits potential distortion from sampling bias; use of out-of-sample, ahead-of-time measures of performance further strengthens generalizability. Use of content and metadata overcomes the limits of either used alone: highly discussed incorrect research or falsely inflated citation counts and major theoretic advances without any secondary spread can be handled by a model incorporating both inputs.

Our demonstration that a purely content-based model, shorn of author and institutional features, is highly predictive of translational impact shows that the predictive signal does not merely reflect institutional productivity or prestige and can be used to address ethical issues associated with reliance on metadata, such as weighting of institutions on purely historical performance. Any individual or institution can submit test data to the model and independently validate predictions over time or retrain with further, prospectively acquired data to ensure adequate handling of future time-varying trends or extension to other data types within MAG, such as preprints. The fidelity of any prediction is inevitably constrained by the quality of the data used to train the model from which it is derived; as bibliographic databases improve, so should the models built on them. Further development might also helpfully include semantic analysis to contextualize the high-dimensional content embeddings, allowing further insights into emerging patterns of translational impact.

Our work builds on existing research on patent, guideline, and policy inclusions. A “patent-paper citation index” has been proposed to formalize science-to-technology linkages,^33^ and patent inclusions have been systematically evaluated to quantify value return on public research investments^34^ and used as a marker of the technological importance of scientific papers.^23^ Although it may seem that patents should precede published research, a large study of United States patent and paper linkage found that 60% referenced prior research.^35^ Patent inclusions have therefore been explored as indicators of papers whose recognition has been delayed^35^ and, therefore, are an established indicator for translational merit, especially of basic science. Similarly, a focus on impact assessments has prompted analyses of referencing patterns within cancer guidelines,^36^ small hand-curated groups of guidelines,^37^ and, separately, policy inclusions extracted by hand,^24^ systematic analysis of coronavirus disease 2019 ( COVID-19) policy,^26^ or from Altmetric,^38^ although difficulties with comprehensive data acquisition have hampered the latter. Although one study has recently attempted to predict combined guideline and clinical trial citations of basic research using a small set of Medical Subject Headings term-derived features,^19^ no comprehensive predictive framework for the tangible product of scientific research, rather than trials, has been described previously. The critique of paper citation metrics for measuring impact is not new and has been described at length elsewhere, but the argument can now be rigorously tested against objective markers of translation.

Application of highly expressive language models to searchable, comprehensive, fully digitized repositories of scientific publications has the power to derive compact but rich representations of research activity on which high-fidelity predictive models can be founded. Here focused on the task of predicting translational signals, the approach can be used to forecast many aspects of scientific activity upstream of real-world impact. Our work argues for a radical shift toward adoption of novel methods for evaluation of medical research, a shift for which observed levels of translational productivity—declining for more than half a century—demand urgent and decisive action.

## Experimental procedures

### Resource availability

#### Lead contact

Further information and requests for resources should be directed to the lead contact, A.P.K.N. (amy.nelson@ucl.ac.uk).

#### Materials availability

This study did not generate new unique reagents.

### Data

The dataset was downloaded from MAG, the largest and widest citation coverage open publications database,^27^^,^^39^ in March 2019. It was filtered to include medical papers, as labeled by MAG, published from January 1990 to March 2019, with at least one paper-to-paper citation. Papers were extracted by filtering for “Doc-Type” attribute “journal”; medical papers were further isolated by filtering on the “Field” code specific to “Medicine.” To extract a patent inclusion count, papers were matched by ID to the reference list on patent entries, in turn provided within MAG through the Lens database; a detailed description of these data sources is available elsewhere.^40^ To extract guideline or policy inclusion counts, papers were matched by title to a dataset kindly provided by the Wellcome Trust, containing reference lists scraped from documents on the World Health Organization, National Institute of Clinical Excellence, UNICEF, Médecins Sans Frontières, United Kingdom government, and United Kingdom Parliament websites. A free web-based tool for guideline and policy inclusion detection is available from Wellcome Data Labs (https://reach.wellcomedatalabs.org/), and associated code is available (https://github.com/wellcometrust/reach). Title matching was by a combination of fuzzy matching and cosine similarity of term frequency-inverse document frequency vectors, with manual cleaning of the resulting matches focused on titles with low fuzzy matching and cosine similarity scores and shorter word counts.

The full feature list extracted from MAG is included in Table S2 and summarily comprised publication year; paper citation count; paper rank; author count; reference count; and rank, paper count, and paper citation information for the first and last author, the first and last authors’ affiliations, the journal, and the field. First and last authors were isolated from ordered author lists supplied in MAG and used in place of the full author list to avoid variably sized or sparse author feature sets, with the rationale that these authors tend to have the largest effect on a paper. The first level of medical domain fields were extracted, 43 in total, and added as features using multiple one hot encoding. Field names from hierarchical topic modeling were supplied in MAG,^41^ and rank, a reinforcement learning estimation of dynamic eigencentrality, reflecting a paper’s connectedness to other influential entries in the graph,^42^ was also supplied in MAG. In addition to a simple paper citation count, the number of times a paper was referenced in the text body of another paper was summed to create a “paper mentions” count.

### Predictive analysis

#### Natural language processing

Medical papers were further filtered to include those with titles and abstracts. Sentence-level embeddings were generated for each title using BioBERT,^28^ a state-of-the-art BERT language model pre-trained on biomedical corpora comprising PubMed abstracts and PubMed Central full-text articles, in addition to general corpora comprising English Wikipedia and BooksCorpus. BERT is a highly influential Transformer encoder, released in 2018, that is able to learn the context of words by joint conditioning on the full sentence rather than creating a sequential representation where context is lost with increasing distance between words.^43^ The sentence-level embeddings were derived from the output of the first (classification) token.

To create a fixed-length abstract-level embedding, we truncated the abstracts to 20 sentences or zero padded where the abstract was shorter, replacing each sentence with its BioBERT embedding and concatenating the array to create a 15,360-dimensional vector. The truncation threshold was motivated by empirical investigation of abstract sentence count distribution within training data; 92% of papers had 20 sentences or less (Figure S7). This was further concatenated with the title vector, creating a 16,128-dimensional representation of the title and abstract taken together.

#### Preprocessing

To rebalance the proportions of positive and negative target labels, the majority negative class was randomly sampled without replacement; this rebalancing strategy was motivated by the abundance of data, a preference toward fewer assumptions at the cost of poorer fit, and the desire to avoid linear oversampling techniques, such as synthetic minority oversampling, which have been shown to underperform in higher dimensions.^44^ Papers without a title or abstract were then removed. This led to a 1.1:1 balance of positive to negative labels in the patent group and the guideline or policy group. Data were randomly split into label-stratified training and test sets with a 9:1 ratio. Missing values in the metadata were imputed with medians derived from the training split, and values were transformed into *Z* scores.

#### Modeling

To address the primary objective of detecting signals of translation, we trained a series of models to predict a binary outcome of inclusion in a patent versus none and, separately, a binary outcome of inclusion in a guideline or policy document versus none. This was motivated by two considerations: first, that each outcome was an independent measure of translation rewarding predominantly fundamental, basic science or applied, clinical (and meta-analytical) science in patent and guideline or policy classes, respectively, and second, that patent inclusions were around 50 times more prevalent than guideline or policy inclusions and might unfairly dwarf the predictive signal of the latter class.

We first modeled a single variable—paper citations per year—using logistic regression to provide a baseline prediction reflecting current citations-based practice (Citations-LogisticRegression). The hyperparameters of this model were optimized using a parallelized, cross-validated grid search. Logistic regression was selected for its simplicity over hyperparameter-optimized MLPs given statistically equivalent 10-fold cross-validation performance. Second, we modeled the metadata—all features extracted from MAG pertaining to the paper and its research environment, excluding title and abstract embeddings—using a 6-layer perceptron with categorial variables one-hot encoded (Metadata-MLP). Third, motivated by the tiled structure of the numerical abstract and title representations, we trained a 1-dimensional CNN for classification, using an initial kernel length and stride of 768 to match the length of each sentence vector, yielding a compact text representation upstream of the fully connected layers.

These two models were tuned by cross-validation within the training set, and the best models were combined into a final model that took the metadata and title-abstract embeddings as inputs, as specified in Figure S1 (Full-MLP-CNN). The differing tensor sizes of metadata and title-abstract embeddings at the concatenation layer of the final model matched the optimal architectures of the individual models; the need for higher relative compression of the title/abstract embeddings likely reflects the higher density of information in the metadata.

#### Interpretability

Deep neural networks do not explicitly provide quantification of the importance of individual features to prediction. We therefore trained and grid-search-optimized an AdaBoost model^45^ on metadata features (Metadata-AdaBoost), chosen for its explainability balanced with good sensitivity to linear and non-linear effects, and extracted Gini-importance from the best-performing model on validation data.

To illuminate the title-abstract embeddings, we trained a fully connected autoencoder on the 16,128 BioBERT dimensions of each title and abstract, deriving a 50-dimensional representation compressed to two dimensions with t-distributed stochastic neighbor embedding (t-SNE).^46^ The resulting plot was colored by the presence or absence of a translation inclusion.

#### Model evaluation

The predictive performance of all models on the training set was evaluated by stratified 10-fold cross-validation using AUROC and AP, a measure of the area under the precision recall curve. The former is a common metric for assessing predictive performance that balances sensitivity against specificity across a range of classification thresholds, and the latter is more resistant to imbalanced data bias and balances sensitivity against precision, the purity of predicted positive results. The final, tuned, highest-performing model was tested on the unseen test data and assessed by AUROC and AP. All AUROCs and APs were macro averaged.

To assess the performance of the final model on future papers, the same architecture was trained from scratch on data from January 1990 to December 2013 and tested on data from January 2014 to December 2017. Papers published from January 2018 to March 2019 were not used for testing because of the short latency of conversion to first patent, policy, or guideline inclusion (Table S3). We measured the performance in the full set of future papers to obtain summary metrics, in addition to individually across each of the 4 years, and within the top 8 fields to investigate the calibration to these groups. Any papers with multiple field membership were considered in each appropriate field. To quantify any reliance on time-dependent citation patterns for a given paper, we assessed the performance of the full model whose training set had “paper citation count,” “paper rank,” and “paper mentions count” variables removed; similarly, to quantify any reliance on features denoting merit extrinsic to the paper, author-, institution-, journal-, and field-level ranks and counts were removed.

As further validation, an external, publicly available dataset containing the publication output of Nobel Prize laureates in physiology or medicine^47^ was downloaded, matched to MAG, and processed identically to the test data. All papers from 1990 to 2019 published up to and including each prize-winning paper were tested on Citations-MLP, Metadata-MLP, and Full-MLP-CNN patent inclusion models retrained on the entire corpus with the tested papers removed. The AUROCs and numbers assigned to positive and negative labels were recorded. Nobel prizes were counted from 1991–2019 to allow analysis of at least 1 year of papers preceding the first award.

### Descriptive analysis

As a secondary objective, we sought to understand the correspondence of patent and guideline or policy citations to the far more widely measured and acknowledged paper citations as well as to understand the community structure of patent included versus non-included groups and guideline or policy included versus not included groups.

Toward the former aim, we plotted paper citations against translation inclusions and examined their correlation by fitting a linear regression model with 1,000× bootstrapped confidence intervals. We ranked journals by paper citation counts normalized by the journal’s total paper count within our dataset, filtered as described for medical papers from 1990–2019 with at least one citation. This roughly corresponds to a canonical “impact factor,” although the interval is widened from yearly to three decades. We repeated this for a journal’s patent inclusions count and guideline or policy inclusion count. Journals analyzed in this manner were filtered to include only those with 500 or more total papers in the dataset.

Toward the latter aim, we fit Bayesian weighted, non-parametric, nested stochastic block models^29^ on all papers with patent inclusions and all papers without them and then again on all papers with guideline or policy inclusions and all papers without them, degree corrected and weighted exponentially by the absolute value of the pairwise correlations of features extracted from MAG (excluding titles and abstracts). Stochastic block models are generative random graph models that display community structures, subsets of nodes connected by larger edge densities than those outside of the subset. The models were strengthened by sampling from the posterior distribution and equilibrated with Markov chain Monte Carlo over 100,000 iterations to ensure convergence. Scalable force-directed placement^48^ was used for visualization of the combined feature graph, with node size proportional to eigencentrality and edge weight and color proportional to the absolute value of the correlation coefficient between two features.

#### Analytic environment

All analyses were written in Python 3.5. Preprocessing was performed using Pandas,^49^ NumPy,^50^ and Scikit-Learn^51^ and visualization using Matplotlib,^52^ Seaborn,^53^ and Graph-tool.^54^ Neural networks were built in Keras^55^ with Tensorflow backend and PyTorch;^56^ other models were built in Scikit-Learn. t-SNE was performed using Multicore-TSNE,^57^ and BioBERT models were downloaded and implemented locally. The hardware specification used was as follows: 96 gigabyte random access memory, Intel Xeon(R) central processing unit E5-2620 v.4 at 2.10 GHz × 32 processor, and GeForce GTX 1080/PCIe/SSE2 graphics.

## Data Availability

This paper analyzes existing, publicly available data, available by application to MAG (https://www.microsoft.com/en-us/research/project/microsoft-academic-graph/). Guideline and policy data are available from the Reach project at Wellcome Data Labs (https://reach.wellcomedatalabs.org/). Code for extracting guideline and policy references is available at https://github.com/wellcometrust/reach. Analytic code will be made available upon reasonable request. Any additional information required to reanalyze the data reported in this paper is available from the lead contact upon reasonable request.

## Supplemental information

Document S1. Figures S1–S8 and Tables S1–S3

Document S2. Article plus supplemental information
