# Supplementary material for: Deep forecasting of translational impact in medical research
Source: Patterns (N Y). 2022 Apr 8;3(5):100483. doi: 10.1016/j.patter.2022.100483 (PMC9122964; doi:10.1016/j.patter.2022.100483)
Supplement: Document S1. Figures S1–S8 and Tables S1–S3 [file mmc1.pdf]

Patterns, Volume *3*

Supplemental information

Deep forecasting of translational impact in medical research

Amy P.K. Nelson, Robert J. Gray, James K. Ruffle, Henry C. Watkins, Daniel Herron, Nick Sorros, Danil Mikhailov, M. Jorge Cardoso, Sebastien Ourselin, Nick McNally, Bryan Williams, Geraint E. Rees, and Parashkev Nachev

Supplemental Materials

Supplemental Figures

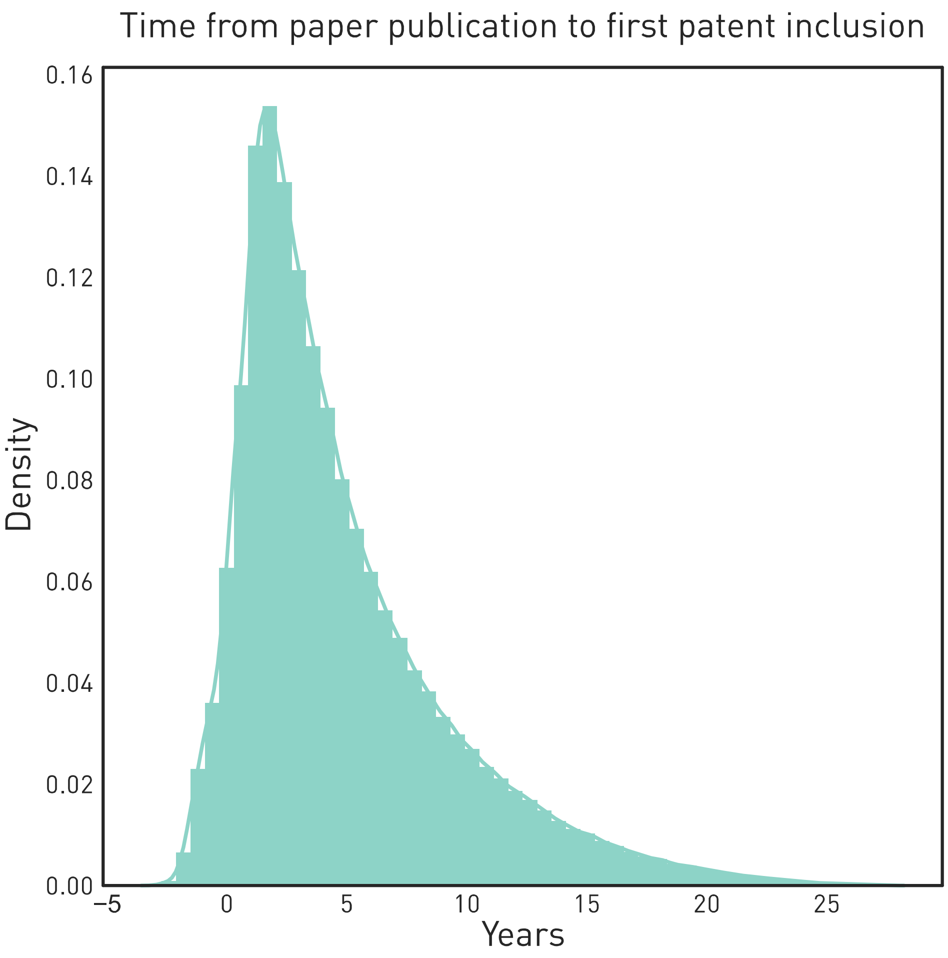

Fig. S1. Distribution of time delay from paper publication to first patent inclusion in years. The mean time until first patent inclusion is 4.73 years (standard deviation 4.54). A small group of negative time delays are present due to variations in listed print and online publication dates within MAG. The x-axis has been narrowed for clarity.

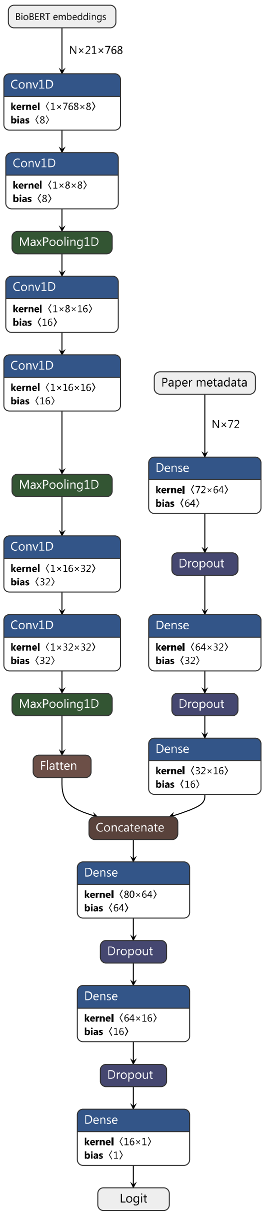

Fig. S2. Network architecture for the combined paper metadata and title/abstract embeddings model. A convolutional decomposition of the abstract BioBERT embeddings is combined with a fully connected model of the metadata to yield a classification of patent or guideline inclusions. See text for full details.

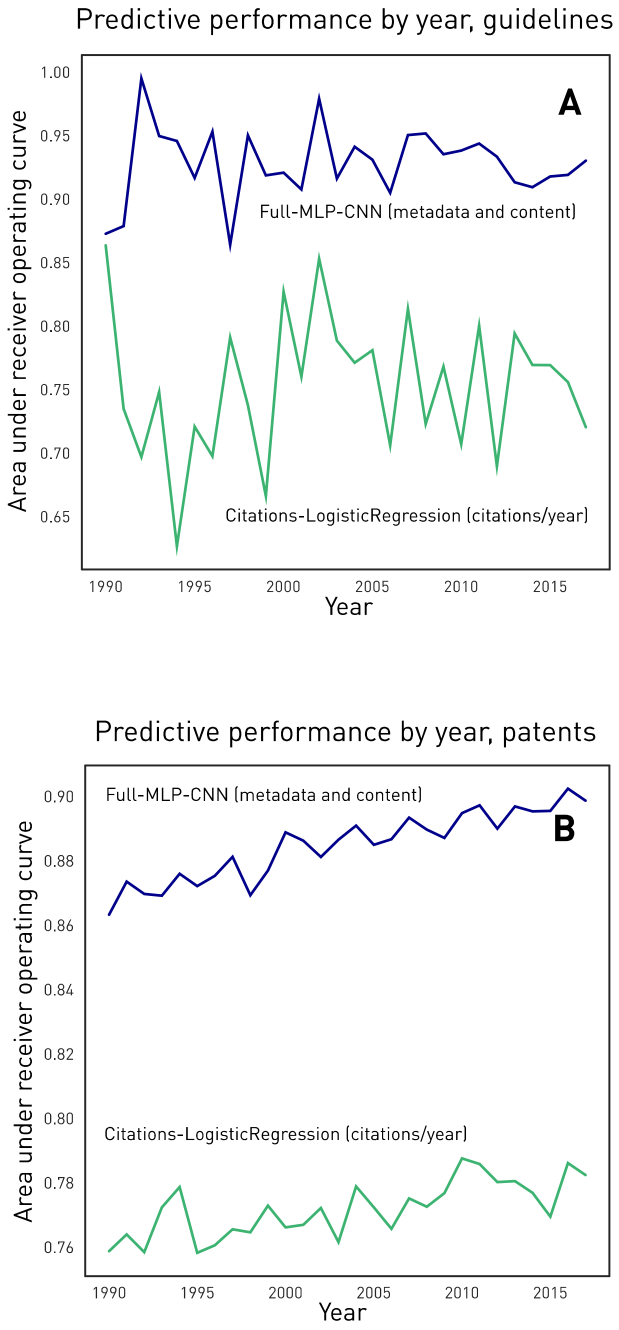

Fig. S3. Predictive performance of Full-MLP-CNN and Citation-LogisticRegression models by AUROC over time (year), for guideline or policy inclusion (A) and patent inclusion (B). Note the y-axis has been narrowed for clarity.

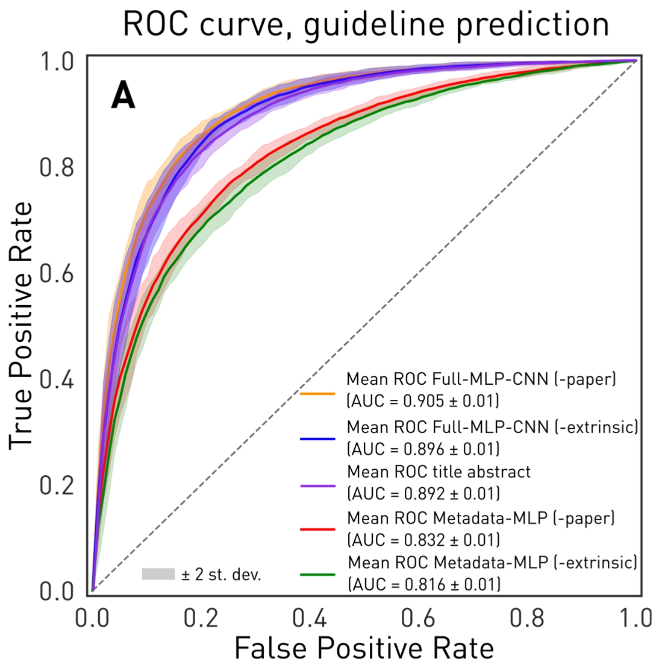

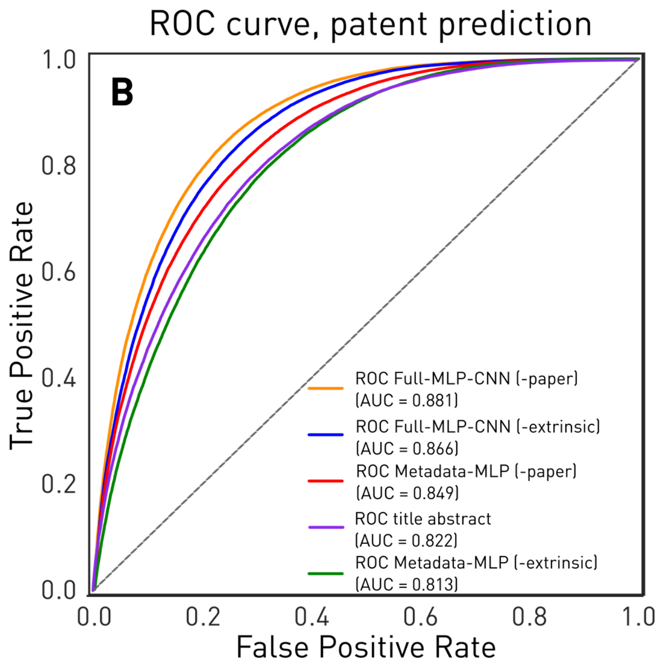

Fig. S4. Model predictive performance with restricted training data. (A) Cross-validated ROC curves for guideline or policy inclusion prediction, (B) ROC curves for patent inclusion prediction. Hybrid model trained on title-abstract embeddings without paper citation features (orange), and also without extrinsic features (blue); metadata-only model without paper citation features (red), and also without extrinsic features (green); and model of only title-abstract embeddings (purple). Confidence intervals are ± 2 standard deviations on 10-fold cross-validation.

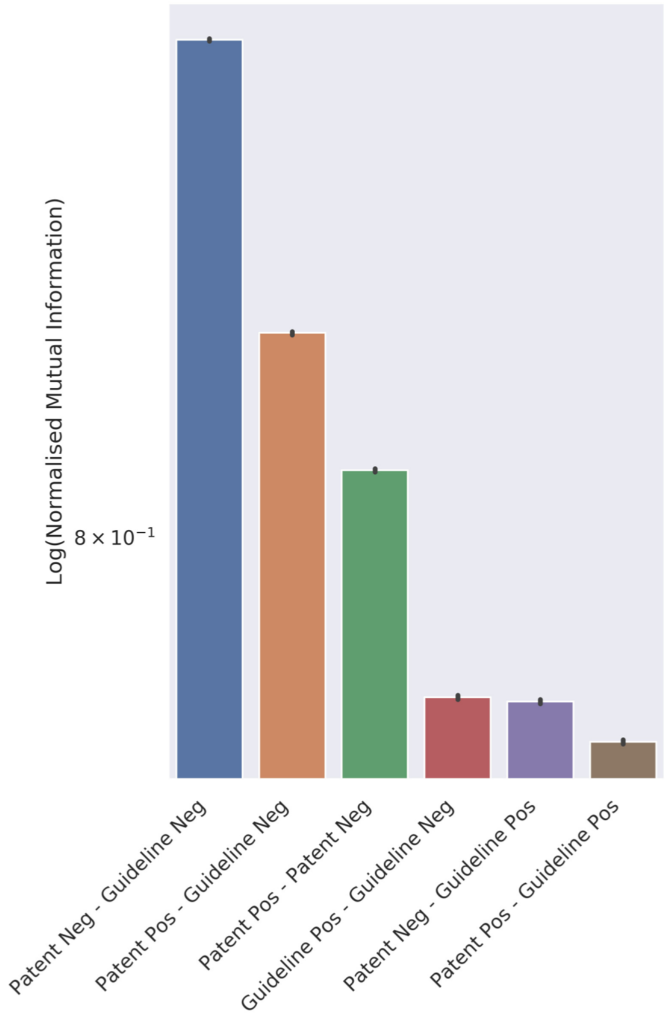

Fig. S5. Log normalised mutual information plotted by the difference between patent inclusion positive and negative, and guideline or policy inclusion positive or negative groups.

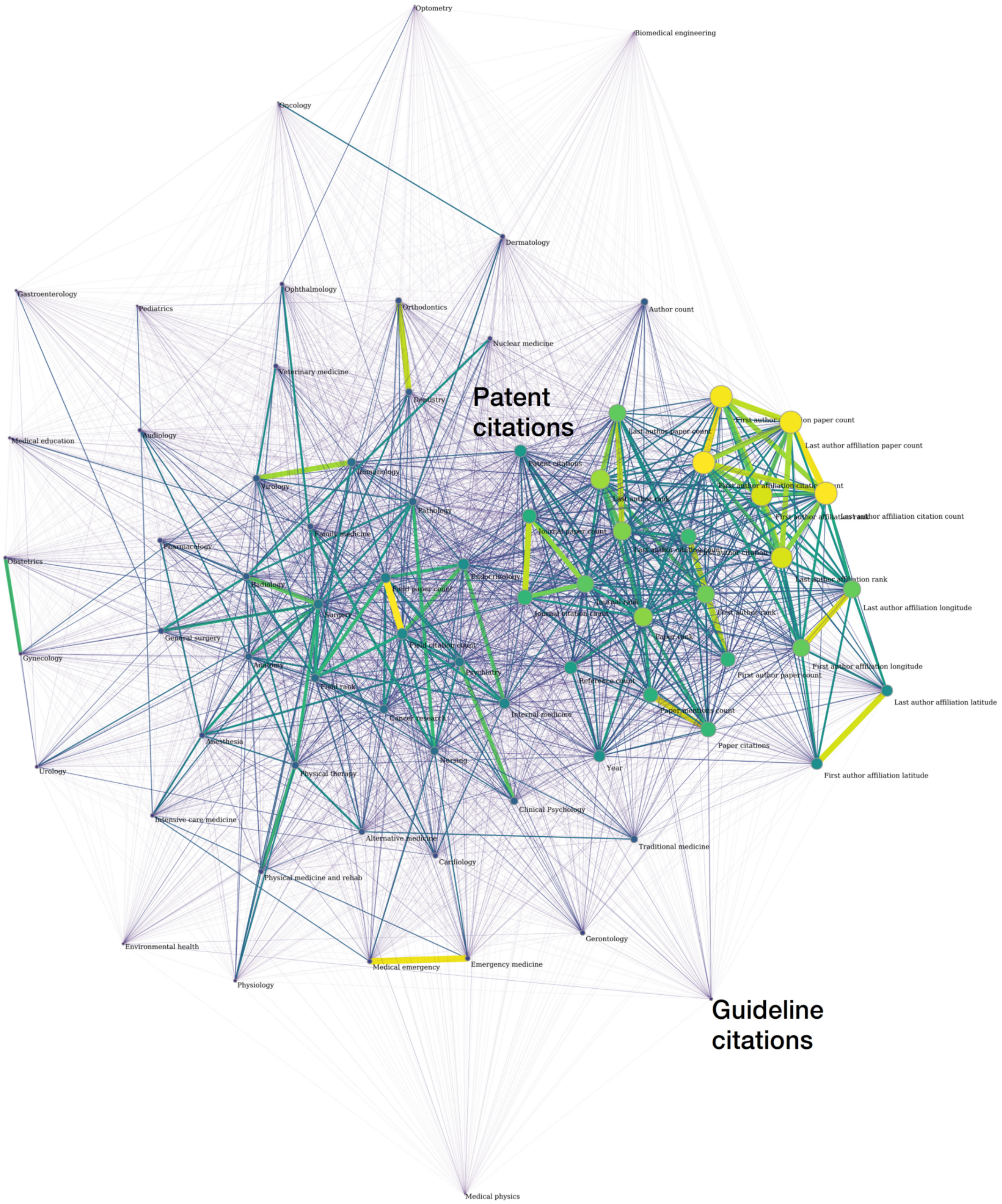

Fig. S6. Scalable force directed placement of the combined features graph, with node size proportional to eigencentrality, and edge weight and colour proportional to the absolute value of the correlation coefficient between two features.

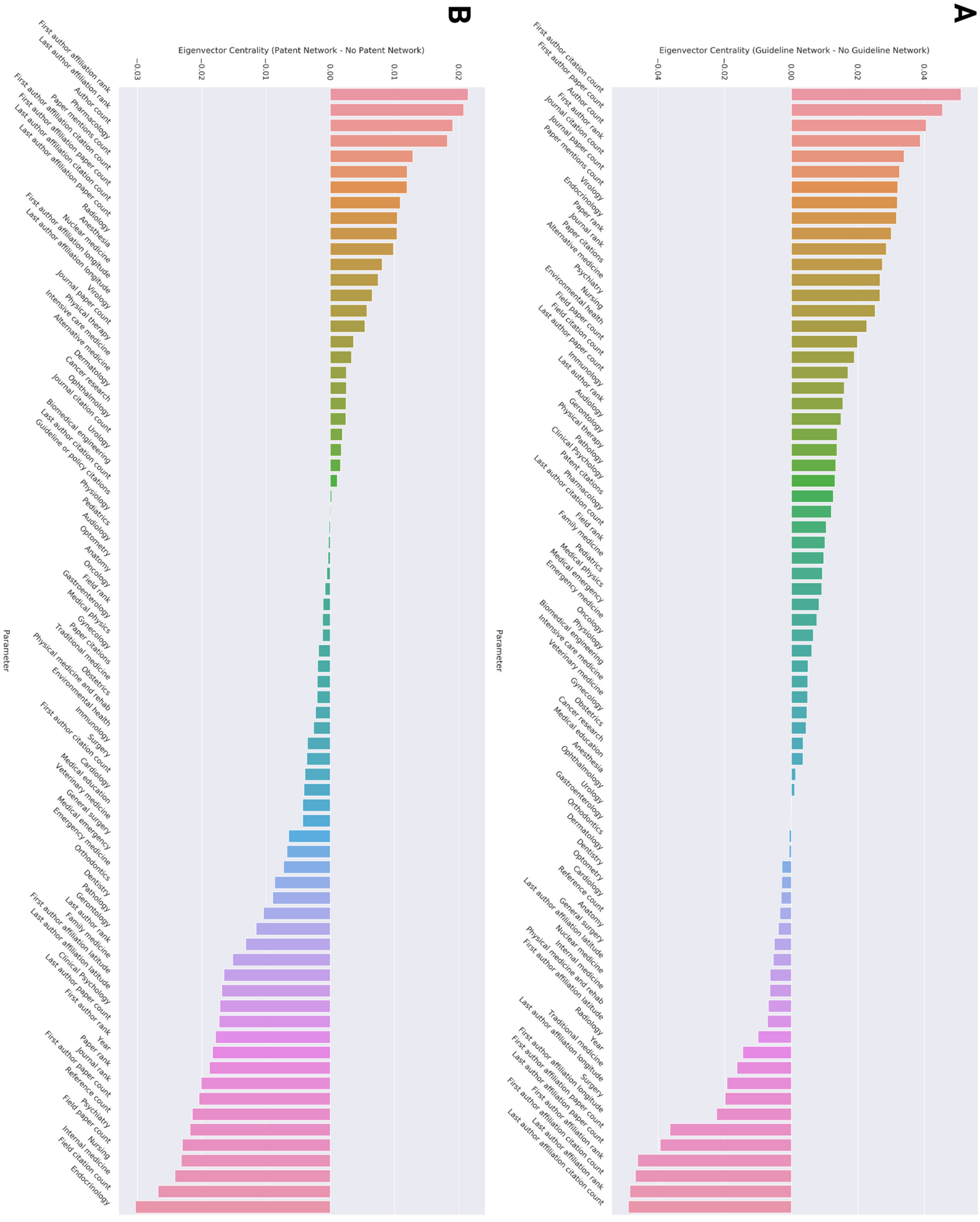

Fig. S7. The subtracted eigencentralities of metadata features for papers included in guidelines or policy vs and those not (A). The subtracted eigencentralities of metadata features for papers included in patents vs those not (B). Values were z-score standardised.

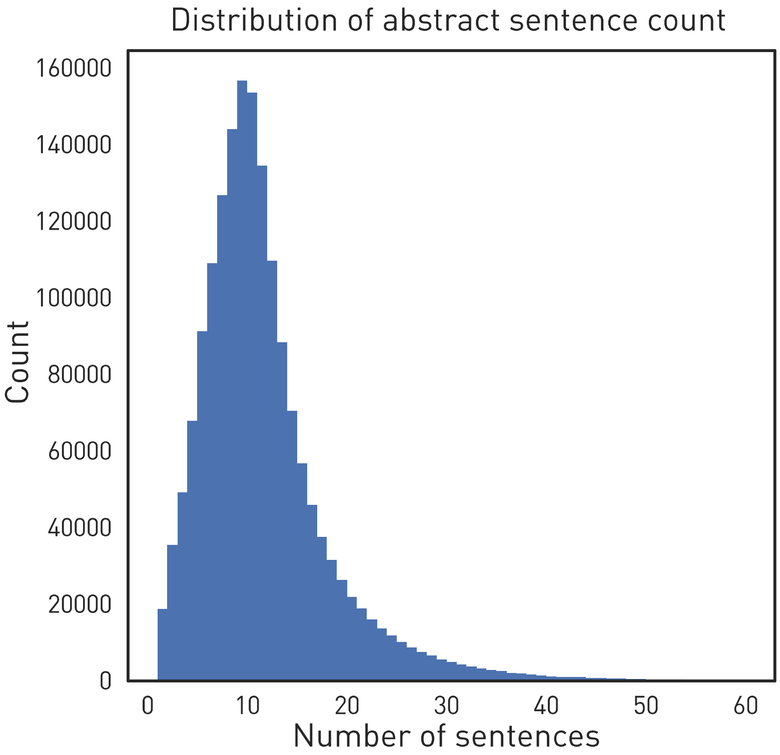

Fig. S8. Distribution of abstract sentence length in analysed papers. The mean sentence count is 11.25 (standard deviation 8.32). The x-axis has been narrowed for clarity.

Supplementary Tables

Table S1A. Top 10 feature importances from AdaBoost model trained on metadata from 1990-2019 for guideline or policy inclusions (left) and patent inclusions (right).

Guideline or policy variable	Gini-importance	Patent variable	Gini-importance
Paper rank	0.155	Journal citation count	0.176
Journal paper count	0.085	Journal rank	0.146
Journal rank	0.070	Journal paper count	0.125
Journal citation count	0.055	Citations	0.087
First author affiliation longitude	0.045	Field paper count	0.049
Citations	0.040	Year	0.045
First author rank	0.025	Paper rank	0.036
Nursing field	0.025	Field rank	0.023
First author affiliation paper count	0.025	Last author affiliation latitude	0.019
Internal medicine field	0.020	Immunology field	0.018

Table S1B. Top 10 feature importances from AdaBoost model trained on metadata from 1990-2013 for guideline or policy inclusions (left) and patent inclusions (right).

Guideline or policy variable	Gini-importance	Patent variable	Gini-importance
Paper rank	0.135	Citations	0.180
Journal paper count	0.070	Field paper count	0.120
Journal rank	0.065	Year	0.075
Journal citation count	0.055	Paper rank	0.060
First author affiliation longitude	0.045	Journal rank	0.055
Citations	0.045	Journal citation count	0.055
First author affiliation latitude	0.035	Paper mentions	0.045
First author rank	0.025	Immunology field	0.045
Nursing field	0.025	Field rank	0.030
Last author affiliation paper count	0.025	Reference count	0.025

Table S2. Metadata feature list

Feature	Categorical (Y/N)	Feature	Categorical(Y/N)
Year	N	Dentistry field	Y
Reference count	N	Dermatology field	Y
Paper citations	N	Emergency medicine field	Y
Paper rank	N	Endocrinology field	Y
Paper mentions	N	Environmental health field	Y
Author count	N	Family medicine field	Y
First author rank	N	Gastroenterology field	Y
First author paper count	N	General surgery field	Y
First author paper citations	N	Gerontology field	Y
First author affiliation rank	N	Gynecology field	Y
First author affiliation paper count	N	Immunology field	Y
First author affiliation paper citations	N	Intensive care medicine field	Y
First author affiliation longitude	N	Internal medicine field	Y
First author affiliation latitude	N	Medical education field	Y
Last author rank	N	Medical emergency field	Y
Last author paper count	N	Medical physics field	Y
Last author paper citations	N	Nuclear medicine field	Y
Last author affiliation rank	N	Nursing field	Y
Last author affiliation paper count	N	Obstetrics field	Y
Last author affiliation paper citations	N	Oncology field	Y
Last author affiliation longitude	N	Ophthalmology field	Y
Last author affiliation latitude	N	Optometry field	Y
Field rank	N	Orthodontics field	Y
Field paper count	N	Pathology field	Y
Field citation count	N	Pediatrics field	Y
Journal rank	N	Pharmacology field	Y
Journal paper count	N	Physical therapy and rehabilitation field	Y
Journal citation count	N	Physical therapy field	Y
Alternative medicine field	Y	Psychiatry field	Y
Anatomy field	Y	Radiology field	Y
Biomedical engineering field	Y	Surgery field	Y
Anesthesia field	Y	Traditional medicine field	Y
Audiology field	Y	Urology	Y
Cancer research field	Y	Veterinary medicine field	Y
Cardiology field	Y	Virology field	Y
Clinical psychology field	Y		

Table S3. Guideline or policy inclusion, and patent inclusion counts and proportions in years 2014-2019. There is a marked drop-off in positive target labels in later years due to the reduced proportion of papers reaching their first translation inclusion within a diminishing timeframe.

Year	Guideline or policy inclusion count	Guideline or policy inclusion %	Patent inclusion count	Patent inclusion %
2014	996	55.7	10551	21.2
2015	801	50.0	6332	13.6
2016	589	43.8	2139	5.4
2017	368	36.7	352	1.1
2018	132	30.4	50	0.3
Jan-Mar 2019	5	20.8	4	0.3
